# Procedural Success and Clinical Outcomes in Patients Undergoing Percutaneous Coronary Intervention of Anomalous Coronary Arteries

**DOI:** 10.1016/j.jscai.2022.100564

**Published:** 2022-12-19

**Authors:** Anoop N. Koshy, Kartik R. Kumar, Gennaro Giustino, Samantha Sartori, Rebecca Fisher, Vishal Dhulipala, Suvruta Iruvanti, Javed Suleman, Samin K. Sharma, Annapoorna S. Kini

**Affiliations:** a The Zena and Michael A. Wiener Cardiovascular Institute, Icahn School of Medicine at Mount Sinai, New York, New York; b Department of Cardiology and The University of Melbourne, Austin Health, Melbourne, Australia

**Keywords:** anomalous coronary artery, atherosclerosis, coronary anomalies, myocardial infarction, percutaneous coronary intervention, revascularization

Anomalous coronary arteries (ACAs) are rare, with a reported prevalence of 0.2% to 1.2% in patients undergoing percutaneous coronary intervention (PCI).^1^^,^^2^ The unusual location and course of ACAs can prevent selective cannulation and co-axial alignment and guide catheter support, thereby increasing the technical complexity of undertaking PCI in these patients.^3^^,^^4^ although previous work has focused on types of coronary anomalies and strategies for diagnostic catheterization in this population, the outcomes after PCI has been largely limited to case series of patients.^3^^,^^4^ We sought to report the procedural success and clinical outcomes in patients who underwent PCI to ACAs using a large institutional database.

Consecutive patients who underwent PCI at a tertiary hospital between January 2012 and December 2019 were prospectively enrolled in the institutional catheterization laboratory registry and assessed for inclusion in this study. Patients provided informed consent for anonymized data collection and systematic follow-up. Baseline clinical, angiographic, and laboratory data were systematically obtained using standardized forms at the time of index hospitalization for PCI. Clinical follow-up was conducted for all patients by trained research personnel for up to 1 year after PCI.

ACA anatomy was confirmed by 2 independent interventional cardiologists (A.N.K. and K.R.K.) and divided into anomalous right coronary artery (RCA) from the left sinus of Valsalva and anomalous left coronary artery (LCA) from the right sinus of Valsalva or RCA. We sought to first compare outcomes between patients undergoing PCI to ACA (ACA-PCI group) and non-ACA (non-ACA-PCI group). We then compared outcomes between those undergoing PCI to anomalous RCA vs anomalous LCA. The primary end point of the study was a comparison of overall procedural success and a composite of all-cause death, myocardial infarction (MI), and target vessel revascularization (TVR) at 1 year after PCI. MI was defined as per the third universal definition and TVR as any repeated revascularization of any segment within the entire major coronary vessel proximal and distal to a target lesion.

During the study period, 25,038 patients underwent PCI, of which 275 (1.1%) unique angiography reports showed an ACA. Among these, 186 underwent PCI to a non-ACA and 90 (0.36%) to an ACA. The ACA-PCI cohort included 55 patients (61.1%) with an anomalous RCA from the left coronary cusp and 35 (38.9%) with an anomalous left circumflex or left main from the right coronary cusp or RCA. On angiographic evaluation, all patients exhibited atherosclerotic coronary artery disease (CAD), with no patients undergoing PCI for an ectopic intramural course.

When compared with the non–ACA-PCI cohort (n = 24,673), the ACA-PCI cohort demonstrated a trend to being younger (mean age, 64.4 vs 66.4 years; *P* = .09) and were more likely to have a history of previous PCI (54 [60.0%] vs 9587 [38.9%]; *P* < .001). Clinical presentation included stable ischemic heart disease and acute coronary syndromes in 58.6% and 41.4% of the patients, respectively, with no differences between the groups. Most of the patients underwent PCI by femoral access, with drug-eluting stents placed. The overall complexity of CAD as per the SYNTAX score (13.4 ± 10.4 vs 10.0 ± 7.2) and rates of bifurcation PCI (18.2% vs 10.0%) were higher in the non–ACA-PCI cohort (*P* < .05). Despite no significant differences in lesion lengths, patients who underwent ACA-PCI demonstrated a trend to having longer stents implanted (57.1 vs 46.0 mm; *P* = .07).

Procedural success was similar in both groups (ACA vs non-ACA: 92.2% vs 94.9%; *P* = .25). The primary composite outcome of death, MI, and TVR was also not significantly different between the ACA and non–ACA-PCI groups (8 [13.6%] vs 2086 [11.8%]; hazard ratio [HR], 1.05; 95% CI, 0.52-2.10) or the individual components of the primary outcome (Table 1). In particular, no differences in rates of TVR were noted when comparing ACA with the non–ACA group. However, fluoroscopy time (median, 25.2 [17.3-39.0] vs 18 [12.3-26.3]; *P* < .001) and contrast volume (175.4 ± 78 vs 146.4 ± 63; *P* < .001) were significantly higher in the ACA-PCI group than in the non–ACA-PCI group. Moreover, the rates of contrast-induced nephropathy (serum creatinine rise >0.3 mg/dL or ≥50% increase in baseline serum creatinine) were higher in the ACA-PCI group (13.4% vs 7.0%; *P* < .001) than in the non–ACA-PCI group. No significant differences were noted when comparing outcomes in patients who underwent PCI to an anomalous RCA with those to an anomalous LCA (Table 1).

**Table 1:** Adverse events at 1 year after index procedure between ACA-PCI and non–ACA-PCI groups and between LCA and RCA groups.

	ACA-PCI group, n = 90	Non–ACA-PCI, n = 24,673	HR (95% CI)	*P*	ACAs		HR (95% CI)	*P*
					RCA group, n = 54 (60.0%)	LCA group, n = 36 (40.0%)		
All-cause death	2 (3.1)	505 (2.8)	1.10 (0.28-4.43)	.889	1 (2.9)	1 (3.6)	0.62 (0.04-9.93)	.736
MI	0 (0.0)	388 (2.2)	–	–	0 (0.0)	0 (0.0)	–	–
TVR	6 (10.7)	1427 (8.4)	1.15 (0.52-2.57)	.731	4 (11.6)	2 (8.9)	1.25 (0.23-6.81)	.800
Death or MI	2 (3.1)	840 (4.7)	0.66 (0.17-2.64)	.557	1 (2.9)	1 (3.6)	0.62 (0.04-9.93)	.736
Death, MI, or TVR	8 (13.6)	2086 (11.8)	1.05 (0.52-2.10)	.89	5 (14.3)	3 (12.2)	1.03 (0.25-4.31)	.969
All bleeding	4 (4.4)	1044 (4.6)	1.06 (0.40-2.82)	.909	–	–	–	–

Values are n (%) unless otherwise noted. The percentages represent Kaplan-Meier rates at 12 months after index procedure.

ACA, anomalous coronary artery; HR, hazards ratio; LCA, left coronary artery; MI, myocardial infarction; PCI, percutaneous coronary intervention; RCA, right coronary artery; TVR, target vessel revascularization.

This study reports the procedural success and 1-year cardiovascular outcomes in patients undergoing PCI to an ACA. It should be noted that all patients in this study had angiographic evidence of coronary atherosclerosis, which should be distinguished from cohorts of typically younger patients with an intramural proximal coronary course, where diagnostic and therapeutic options vary significantly.^5^ This single-centre experience, to our knowledge, represents the largest cohort of patients with ACA who underwent PCI. The high rates of procedural success and no difference in adverse cardiovascular events at 1 year, when compared with those patients who underwent PCI for non-ACA can be reassuring for interventional cardiologists given the procedural challenges encountered in these cases. Notwithstanding, the higher fluoroscopy time, contrast volume, and associated contrast-induced nephropathy are indicative of the technical complexity and challenges associated with performing PCI in these patients.

Strengths of this study include the inclusion of an all-comer patient population who underwent PCI, systematic recording of procedural data and clinical events, and a well-characterized patient population. Limitations include a lack of information about patients who may have been managed medically owing to technical procedural challenges with intervening on ACA. Although this study reports outcomes from a large-volume tertiary center, it is unclear whether these findings can be generalized to centers with lower procedural volumes.

To conclude, PCI of ACAs for atherosclerotic CAD demonstrated high procedural success and similar 1-year outcomes when compared with PCI of non-ACAs. The higher radiation, contrast volume used, and associated rates of contrast-induced nephropathy in patients who underwent ACA-PCI highlights the real-world challenges of undertaking revascularization in these patients.
